# A Case Report of Bilateral Sequential Central Retinal Artery Occlusion in Occult Giant Cell Arteritis: The Eyes Are Windows to Systemic Disease

**DOI:** 10.7759/cureus.93625

**Published:** 2025-10-01

**Authors:** Manpreet Malik, Rashmitha Somagani, Harikrishna Ollala, Ekkudu Rama Kanth

**Affiliations:** 1 Department of Ophthalmology, ESIC Medical College and Hospital, Hyderabad, IND; 2 Medicine, Kamineni Academy of Medical Sciences and Research Centre, Hyderabad, IND

**Keywords:** aion, central retinal artery occlusion (crao), european alliance of associations for rheumatology (eular) classification, giant cell arteritis (gca), occult gca

## Abstract

Giant cell arteritis (GCA) is an inflammatory condition generally affecting medium to large arteries, commonly affecting the carotid artery and its branches. GCA generally presents with systemic symptoms such as temporal headache, jaw claudication, and polymyalgia rheumatica. Occult GCA is a variant where patients present with ocular complaints without any systemic manifestations. We report a case of a 59-year-old male who presented with a sudden, profound, and painless loss of vision in his right eye. Fifteen days later, he presented with a similar loss of vision in his left eye, which had occurred five days before presentation. Ophthalmological examination revealed posterior segment involvement suggestive of central retinal artery occlusion in the right eye. He was lost to follow-up between both presentations, which delayed the systemic evaluation and led to a sequential attack in the left eye. The disease was diagnosed based on raised inflammatory markers such as erythrocyte sedimentation rate (ESR) and C-reactive protein (CRP) with a classic halo sign on temporal artery ultrasound. The systemic treatment is multidisciplinary and must be initiated early to prevent ocular complications.

## Introduction

Giant cell arteritis (GCA) or temporal arteritis is a chronic granulomatous vasculitis affecting medium- to large-sized vessels. GCA is primarily seen in females older than 50 years of age [1]. Granulomatous inflammation, which consists of CD4 T cells, activated macrophages, and multinucleated giant cells, leads to thickening of the inner layer of these medium to large-sized blood vessels, causing luminal compromise [2]. Systemic manifestations of GCA include temporal headache, jaw claudication, constitutional symptoms such as fever, malaise, anorexia, and weight loss. Ophthalmic manifestations usually present as sudden visual disturbance or loss [1]. 

GCA has a propensity for inflammation of the posterior ciliary artery and cilioretinal artery, causing ischemic optic neuropathies, central retinal artery occlusion (CRAO), choroidal ischemic lesions, and cilioretinal artery occlusion [3]. CRAO presents bilaterally in 28-31% of GCA patients [3]. Diagnosis of GCA is based on European Alliance of Associations for Rheumatology (EULAR) criteria, which include symptoms of GCA and investigations such as erythrocyte sedimentation rate (ESR), C-reactive protein (CRP), temporal artery ultrasound, and biopsy [4]. Occult GCA presents with ocular manifestations in patients with no systemic symptoms; however, these patients have raised ESR and CRP [3]. CRAO is an ocular emergency having a poor prognosis with delayed recognition, as recovery can be achieved if treatment is given within 90 minutes of presentation [3]. We report a case of bilateral sequential sudden loss with CRAO secondary to GCA.

## Case presentation

**Figure 1:**
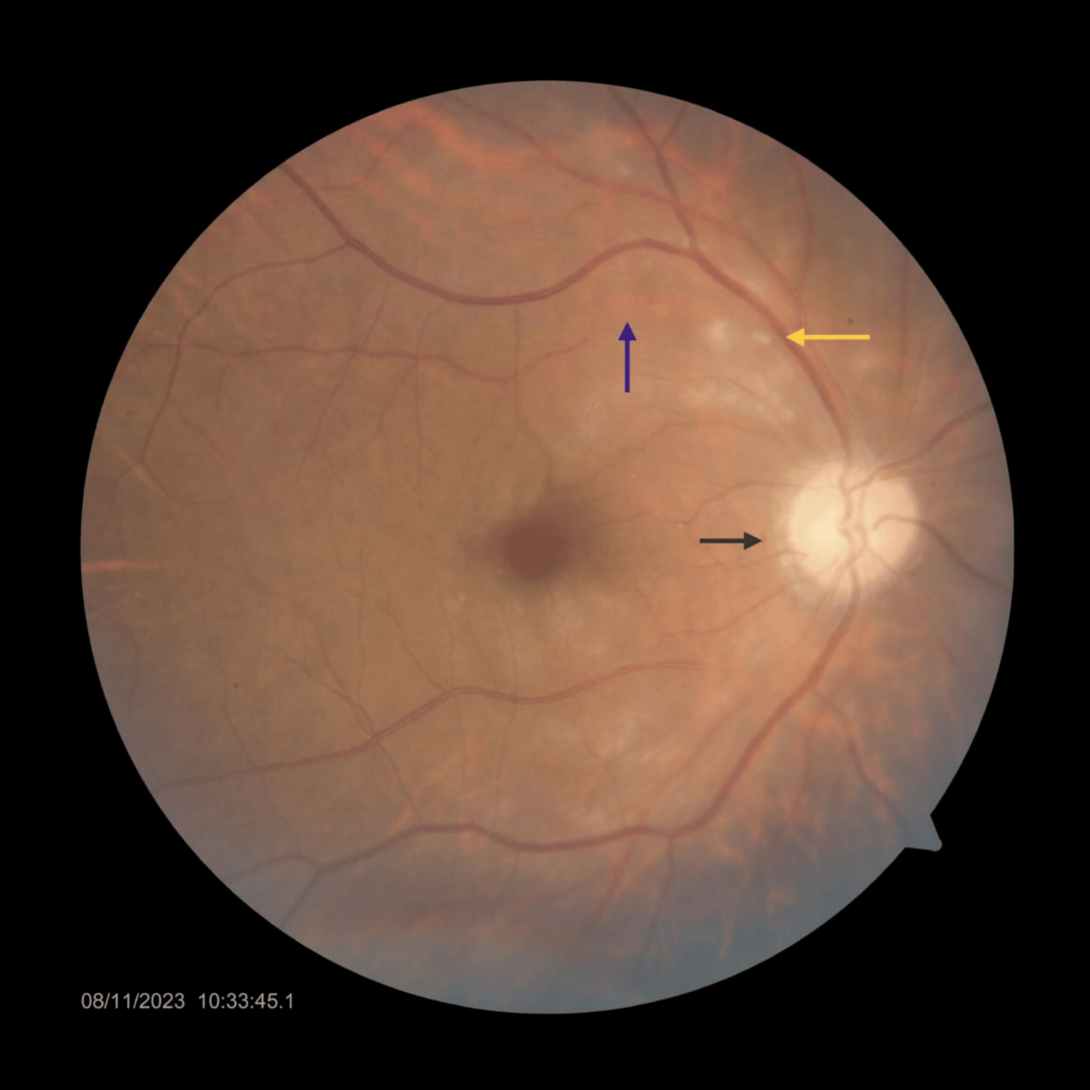
Fundus photo of the right eye The image shows an embolus (blue arrow), multiple superficial exudates (yellow arrow), and a pale optic disc (gray arrow)

The patient was a 59-year-old male who presented to the Ophthalmology Department with a sudden, significant painless decrease in visual acuity in the right eye for one day; he had no notable medical history. An ophthalmologic examination of the right eye revealed a visual acuity of 1.8 log MAR, with an unremarkable anterior segment and no relative afferent pupillary defect (RAPD). Intraocular pressure was within normal limits; the fundus examination showed a pale disc with well-defined margins, and an embolus was noted in the branch of the superior retinal artery with multiple superficial exudates and loss of sheen with blanched appearance of macula (Figure 1). Examination of the left eye was normal.

**Figure 2:**
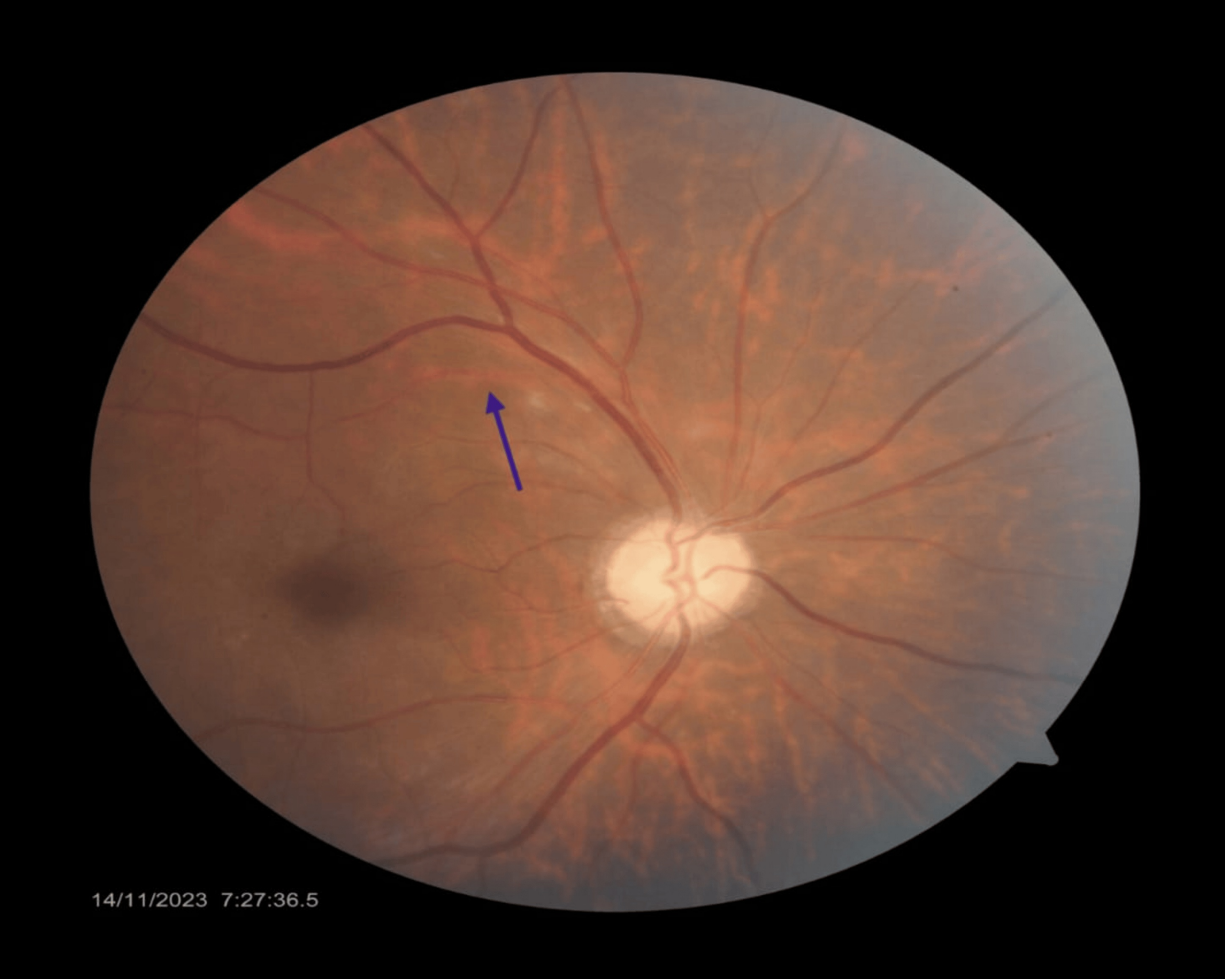
Fundus photo of the right eye The image shows the restoration of reperfusion after the dislodgement of the embolus (blue arrow)

The patient was started on tablet acetazolamide 250 mg, and ocular massage with four mirror goniolens was done for 10 seconds and released for 5 seconds. Ocular massage led to the dislodgement of the embolus, and reperfusion was established (Figure 2). Visual acuity improved to 0.18 logMAR units in the right eye. Systemic evaluation revealed normal carotid Doppler and 2D Echocardiogram (Table 1). Further assessment, which included inflammatory markers and treatment with steroids, was delayed as he was lost to follow-up. 

**Table 1:** Investigations at first presentation ECG: electrocardiogram

Evaluation	Result
Hemoglobin	8.5 gm%
Lipid profile	Normal
Serum homocysteine	11 μmol/ L
ECG	Normal
2D echo	Normal
Carotid Doppler	Normal

**Figure 3:**
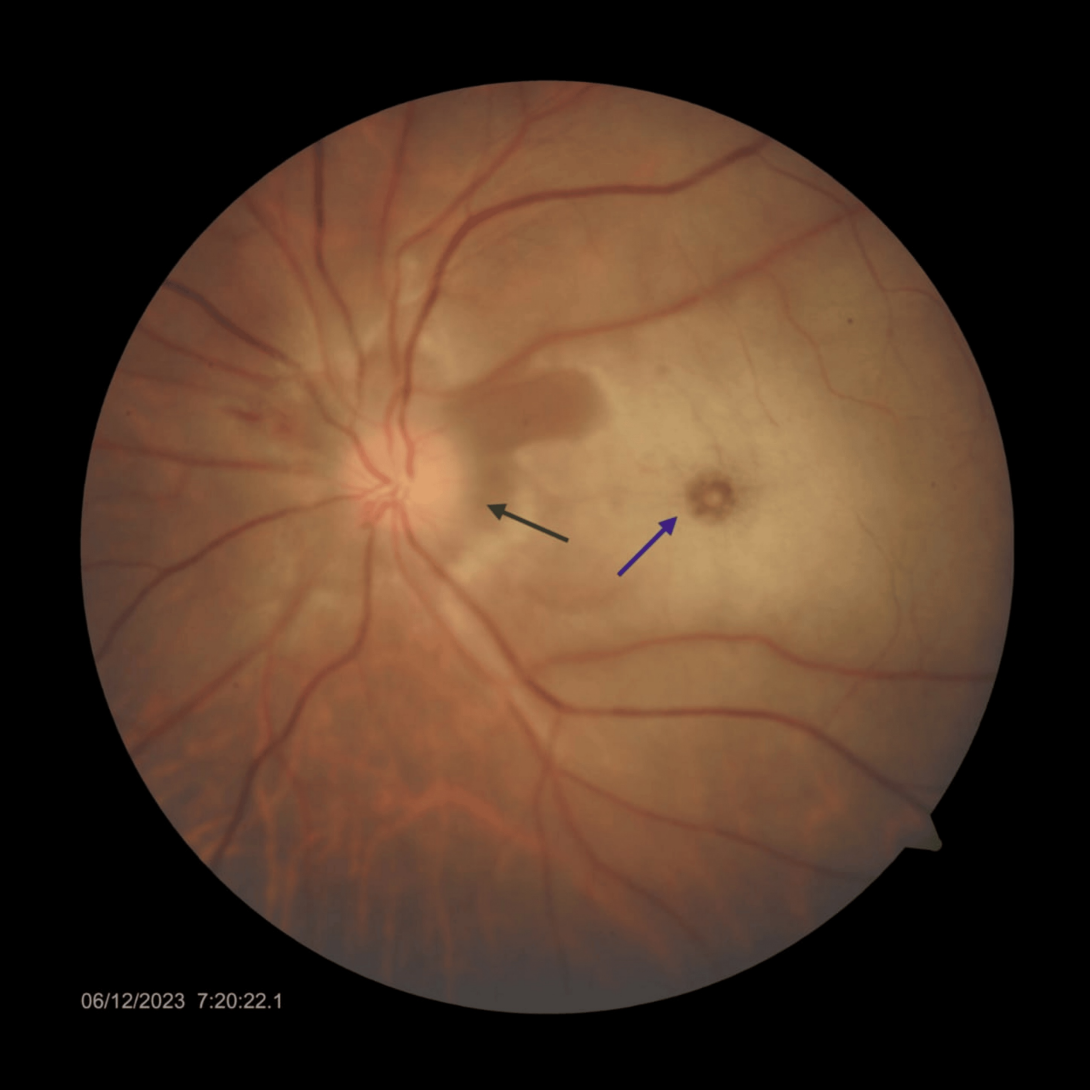
Fundus photo of the left eye The image shows a cherry red spot (blue arrow) and a hyperemic disc with blurred margins (gray arrow)

Fifteen days later, the patient presented with a similar complaint in the left eye for five days. An ophthalmologic examination of the left eye revealed a visual acuity of 2.4 logMAR units, with RAPD grade 2. Intraocular pressure was normal. Fundus examination revealed a pale white retina with a cherry red spot at the fovea, and the optic disc was hyperemic with blurred margins (Figure 3). Fundus fluorescein angiography demonstrated delayed arterial filling, and a diagnosis of CRAO was made. Biological investigations revealed elevated CRP of 12mg/dl and elevated ESR of 82 mm/hr (Table 2).

**Table 2:** Investigations at second presentation CRP: C-reactive protein; ESR: erythrocyte sedimentation rate; USG: ultrasonography

Evaluation	Result
CRP	12 mg/dl
ESR	82 mm/hr
Coagulation profile	Normal
Protein C, S, anti-thrombin	Normal
Lupus anticoagulant	Negative
Rheumatoid factor	Negative
ANA, APLA	Negative
Temporal artery USG	Bilateral wall thickening, halo sign present
Temporal artery biopsy	Negative

**Figure 4:**
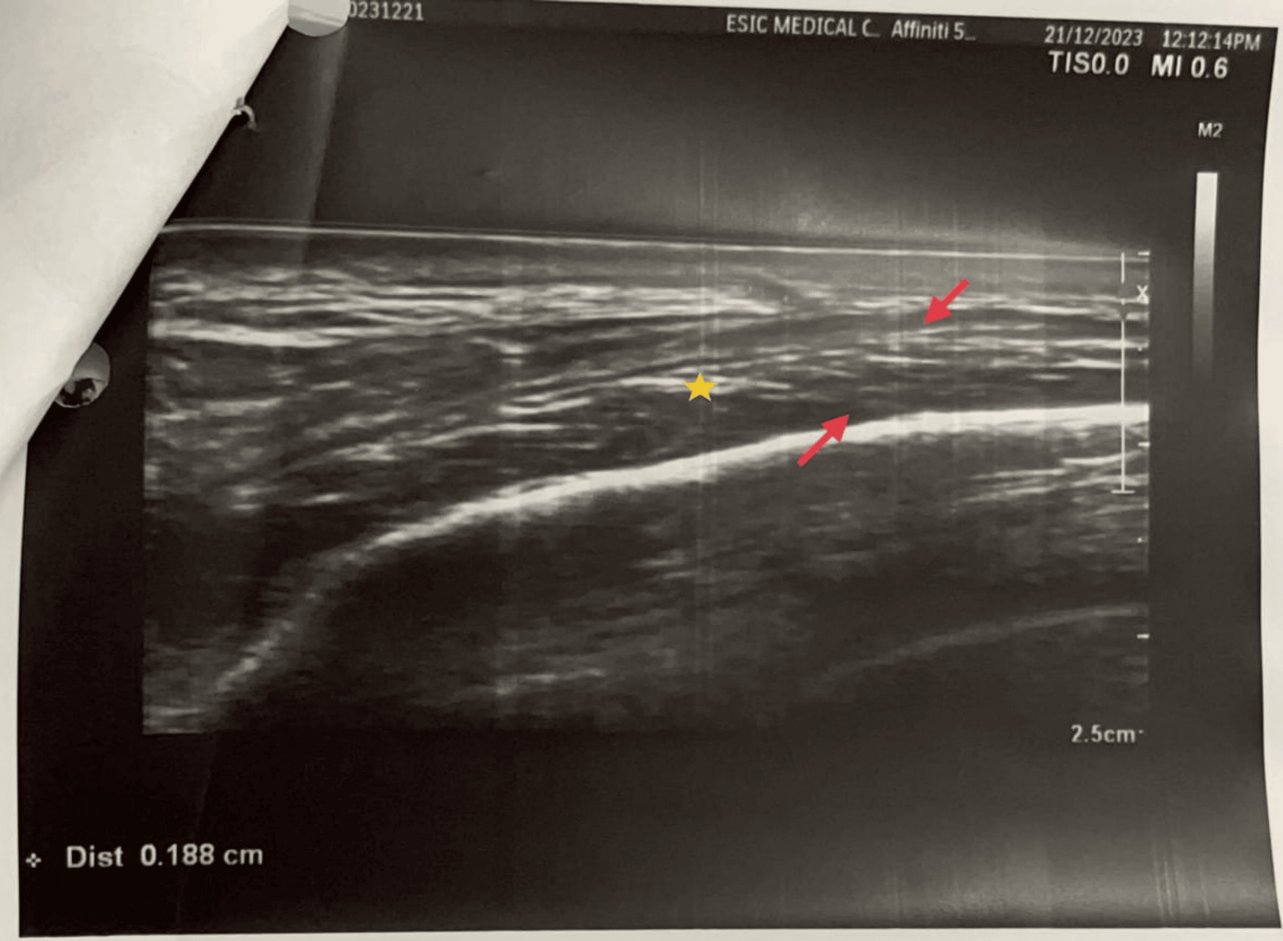
Temporal artery ultrasound The image shows the lumen (yellow star) and the vessel wall (red arrow)

Bilateral temporal artery ultrasound revealed a classic halo sign showing bilateral hypoechoic wall thickening with narrowed calibers (Figure 4). Left temporal artery biopsy came out as negative. Based on the American College of Rheumatology (ACR)/EULAR criteria 2022, the patient was diagnosed with GCA, with a total score of 11 (Table 3).

**Table 3:** EULAR criteria for the diagnosis of GCA A score >6 is positive EULAR: European Alliance of Associations for Rheumatology; GCA: giant cell arteritis; CRP: C-reactive protein; ESR: erythrocyte sedimentation rate; USG: ultrasonography

EULAR criteria	Score
Age >50 years	Absolute criteria
Sudden vision loss	3
ESR >50 mm/hr, CRP >10 mg/dl	3
Halo sign on the temporal artery USG	5
Total score	11

The patient was transferred to internal medicine, where an urgent IV corticosteroid bolus of 1 g/day was administered for three days, followed by an oral corticosteroid regimen of 1 mg/kg/day until normalization of ESR and CRP, and then a gradual tapering. The patient was advised to shift from corticosteroids to immunosuppressants, such as methotrexate, once the anemia improved. Visual acuity on follow-up after one month was 0.18 logMAR in RE and 2.4 logMAR in LE, showing no improvement in LE visual acuity. 

## Discussion

CRAO is an ophthalmic emergency that leads to sudden, profound, and painless loss of vision. The most common cause of CRAO is an embolus that obstructs the retinal artery arising from the carotid artery or the heart. GCA is the second most common cause for CRAO [5]. Bilateral ophthalmic involvement occurs in 28-31% of GCA patients [3]. Patient education about the bilaterality of the disease plays a crucial role in preventing sequential involvement. GCA can present with temporal headache, jaw claudication, fever, anorexia, polymyalgia rheumatica, sudden vision disturbances, and, in some rare cases, tongue necrosis [3]. Occult GCA or silent GCA is an ocular variant characterized by a lack of systemic symptoms. Incidence of occult GCA varies from 14.3 to 21% [6]. Our case also lacked the classical symptoms of GCA and presented with ocular symptoms.

The treatment for CRAO aims at dislodging the embolus or thrombi through various procedures. Ocular massage, anterior chamber paracentesis, oral administration of acetazolamide, inhalation of 95% oxygen and 5% carbon dioxide, rebreathing of expired CO_2_ in a bag, and Nd:YAG embolysis are some procedures to achieve dislodgement [7]. Retinal damage is irreversible if retinal blood flow is not restored within 90 minutes; partial recovery is possible if blood flow is restored within 240 minutes [3]. In our case, vision was restored in the right eye after dislodgement with ocular massage. Vision resortment could have also been due to the presence of the cilioretinal artery. The cilioretinal artery is a branch of the posterior ciliary artery that supplies the macular region. However, no improvement was observed in the left eye due to the late presentation. In our case, further management of the patient, including steroid commencement and inflammatory markers, was delayed as the patient was lost to follow-up.

The diagnosis of GCA is made with blood investigations such as ESR and CRP, a temporal artery ultrasound showing lumen narrowing, and a positive temporal artery biopsy showing granulomatous infiltration of the vessel wall. However, some cases of GCA can reveal a negative biopsy because of the presence of skip lesions. ACR/EULAR 2022 criteria classify a score of more than 6 as diagnostic of GCA. Our patient had a score of 11, and hence was diagnosed with GCA [8] (Table 3). The diagnosis of GCA is challenging but essential, as prompt initiation of systemic corticosteroids can prevent irreversible complications. The treatment starts with high-dose intravenous corticosteroids, followed by high-dose oral glucocorticoids combined with tocilizumab or methotrexate. After clinical remission with controlled ESR and CRP, glucocorticoid dosage can be reduced. If remission is not achieved, an immunosuppressive agent such as abatacept or methotrexate can be used [4].

## Conclusions

GCA is a medical and ophthalmological emergency, where delays can lead to irreversible vision loss in both eyes. The treatment of GCA requires a multi-disciplinary approach, where prompt consultation between the rheumatologist and ophthalmologist is crucial. Diagnosis of CRAO secondary to occult GCA should not be missed; palpation of the bilateral temporal artery and blood investigation, such as inflammatory markers, can help with the diagnosis. Patient education about the severity of the disease, the importance of compliance with treatment, and follow-up is crucial.
